# Correlative Ultrafast Imaging of a Photodriven Phase Transition Using 4D Scanning Transmission Electron Microscopy

**DOI:** 10.1021/acsnano.5c22662

**Published:** 2026-06-08

**Authors:** Arthur Niedermayr, Jianyu Wu, Bertina Fisher, Ido Kaminer, Jonas Weissenrieder

**Affiliations:** † Department of Materials and Nano Physics, School of Engineering Sciences, KTH Royal Institute of Technology, Stockholm SE-100 44, Sweden; ‡ Department of Physics, Technion−Israel Institute of Technology, Haifa 32000, Israel; ¶ Department of Electrical and Computer Engineering, Technion−Israel Institute of Technology, Haifa 32000, Israel; ⊥ Department of Materials Science and Engineering, Technion−Israel Institute of Technology, Haifa 32000, Israel

**Keywords:** ultrafast transmission electron microscopy, ultrafast imaging and strain mapping, phase transitions, four-dimensional scanning transmission electron microscopy (4D STEM), vanadium dioxide

## Abstract

Oxides exhibiting insulator–metal transitions are promising candidates for next-generation ultrafast electronic switching devices. However, critical gaps remain in understanding the onset of strain and its dynamics as these materials undergo structural transitions, particularly in nanostructured configurations. Here, we present ultrafast four-dimensional scanning transmission electron microscopy enabling virtual imaging and strain mapping at every point in space and time. Using this technique, we directly probe a laser-excited phase transition in the prototypical material vanadium dioxide (VO_2_). This direct imaging capability reveals the dynamics of the structural phase transition and connects it to the resulting strain formation on picosecond time scales. We find that the transient in-plane strain reaches ∼1% within ∼20 ps, an order of magnitude larger than expected from thermal expansion of the monoclinic phase. This indicates that the dominant strain contribution originates from the evolving structural phase transformation. Our results reveal the coupling between electronic, structural, and mechanical responses in correlated oxides under nonequilibrium conditions.

## Graphical abstract

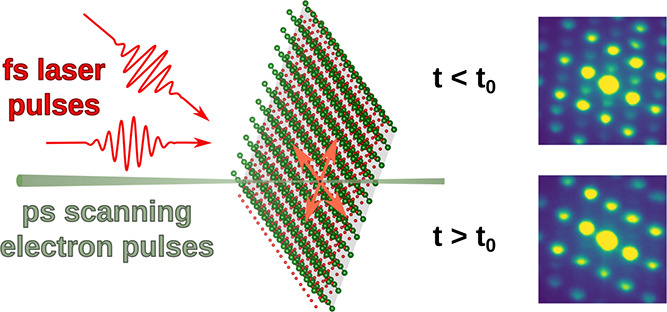

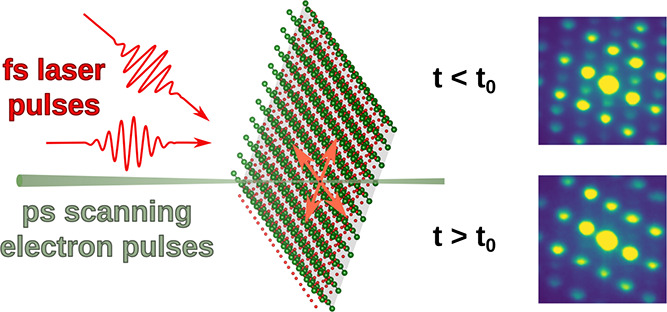

Photodriven insulator-to-metal phase transitions in strongly correlated oxides hold significant potential for next-generation solid-state electronic devices,^1–3^ particularly for ultrafast switching applications. Exploiting the unique properties of these materials, such as their ability to rapidly transition between insulating and metallic states under light excitation, opens the possibility to design devices that operate on picosecond time scales. Their tunable optical and electrical properties make them highly attractive for applications in Mott field-effect transistors, memristive devices, thermal sensors, and chemical sensors.^4–13^ Additionally, the significant changes in reflectivity and conductivity associated with these phase transitions enable the development of ultrafast optical switches and adaptive electronic components.

Photoexcitation with short laser pulses, at high intensities, has in recent years become a central method for studying the underlying behavior of strongly correlated oxides and of other quantum materials. Such short and intense pulse excitations inevitably lead to strain formation.^14^ Understanding the mechanism of strain formation and its influence on phase transitions is a long-standing challenge.^15–17^ Strain can locally modify the lattice structure, directly affecting the temperature, kinetics, and spatial inhomogeneity of the metal–insulator transition, making nanoscale strain mapping crucial to fully understand and control phase behavior. This is particularly important in thin films, where faster switching can be achieved compared to bulk counterparts and where a larger fraction of the material interacts with the light due to reduced screening.

So far, most efforts to understand strain on ultrafast time scales have focused on the generation of acoustic waves, which lead to local sample bending and produce strong contrast. This mechanism has been extensively studied over the past few decades due to its relative ease of detection using bright- and dark-field imaging (or a combination thereof) with local diffractive probing.^18–23^ However, microscopic, local strain dynamics associated with structural phase transitions remain challenging to access and quantify. Despite ongoing efforts, open questions remain regarding the interplay between the structural phase transition and strain on nanometer length scales.

This work explores the mechanisms underlying structural phase transitions induced by pulsed optical excitation. The evolving phase transition generates local strain fields that influence the material and dynamically reshapes the energetic landscape, feeding back into the transition process. To address these limitations and deepen our understanding of the microscopic interplay between strain and phase transitions, we employ direct, quantitative imaging of the spatiotemporal evolution of both strain and structural phase separation. Using the seminal material vanadium dioxide (VO_2_) as a model system, we directly correlate the local phase fraction with lattice strain on picosecond time scales. This approach reveals that the transient strain substantially exceeds that expected from thermal expansion alone, indicating that the dominant contribution to the strain originates from an evolving structural phase transformation.

It is well-established that strain affects the transition temperature of VO_2_ and can be harnessed to tune its electronic and structural properties.^15,24^ Therefore, understanding how acoustic waves and other forms of strain influence performance is key for practical device applications. Notably, strain itself can act as a trigger for the phase transition.^25^ It thus remains a central challenge in the field to distinguish between optically and strain-induced mechanisms in the dynamics of VO_2_.

To address this challenge, we track the intricate evolution of strain during optically induced phase transitions, offering new perspectives on the interplay between electronic excitation, structural changes, and strain at picosecond-nanometer resolution. A key innovation in our approach is the use of a spatially structured optical excitation, which not only induces electronic and structural phase transitions but also provides a means to probe them with high spatiotemporal resolution. The resulting patterned phase separation, characterized by periodic metallic and insulating domains, functions beyond the role of a conventional grating. It enables two critical advancements. First, it introduces optical variations on the scale of hundreds of nanometers, allowing access to dynamic processes at the combined picosecond-nanometer scale. Second, it imposes a well-defined excitation geometry with a known spatial periodicity. Photoinduced responses therefore follow this periodicity, allowing them to be distinguished from static morphology-related contrast arising from specimen thickness variations or bending. In addition, high- and low-fluence regions coexist within the same field of view, enabling direct comparison under identical diffraction conditions. This structured excitation opens up new pathways for precision studies of correlated materials and highlights potential applications in nanoscale optical devices, including energy-efficient transmission gratings for wavelengths below the material’s band gap.

Ultrafast strain dynamics are typically analyzed using ultrafast transmission electron microscopes,^26,27^ where bright- and dark-field imaging is the most commonly employed technique.^18,19,21^ However, these approaches are limited in that they primarily detect strain components aligned with the beam propagation direction because of the strong influence of bending and tilting on the local diffraction conditions. Consequently, achieving quantitative, component-resolved strain mapping on ultrafast time scales remains challenging.

A few pioneering ultrafast diffraction studies addressed this limitation using four-dimensional scanning transmission electron microscopy (4D STEM) with convergent-beam electron diffraction (CBED).^28–30^ These works focused primarily on acoustic strain wave analysis in well-established single-element materials, where strain gradients produce high-contrast diffraction signals. Quantitative strain extraction from CBED patterns typically relies on detailed dynamical diffraction calculations, as the intensity distributions are highly sensitive to crystallographic orientation and strain state.^31,32^ While CBED provides powerful access to nanoscale strain information, it is less straightforward to simultaneously track the evolution of structural phases during ultrafast transitions using Bragg-resolved imaging.

In this work, we implement ultrafast 4D STEM using a nanobeam electron diffraction (NBED) configuration with a small convergence angle, enabling quantitative, component-resolved strain mapping on the nanometer scale. Although the distinction between CBED and NBED is gradual rather than sharp, to our knowledge, the combination of ultrafast strain analysis and Bragg-resolved virtual imaging has not previously been demonstrated. CBED provides high spatial resolution and rich dynamical diffraction information, but quantitative strain extraction typically relies on dynamical diffraction modeling of intensity distributions. In the present NBED configuration, strain is obtained directly from reciprocal lattice vector shifts (Bragg peak positions), enabling a comparatively model-independent determination of the lattice strain. In addition, the 4D STEM data set allows simultaneous postselection of virtual bright-field, dark-field, and other Bragg-resolved imaging modes from the same acquisition, facilitating correlative analysis of phase and strain. In this sense, NBED provides a complementary approach to that of CBED.

Our experimental setup is shown in Figure 1a. In a pump–probe configuration, a pulsed laser is reflected off a metallic mirror^33^ and overlaps with the direct beam on the specimen, generating a transient optical grating through self-interference. A delayed electron pulse is rastered across the specimen, generating a local diffraction pattern at each probe position. This approach enables investigation of the local structural response to a well-defined spatially structured excitation within an otherwise uniform specimen. The specimen’s response to a spatially patterned laser pulse is shown in Figure 1b. Photon absorption in vanadium dioxide induces an electronic and structural phase transition, where illuminated regions of the sample transition from the monoclinic (M1) phase to the rutile (R) phase.^34,35^ The combination of the structural phase transition, the heat gradient, and the resulting strain generates stress gradients and a strain mismatch. These effects induce a mechanical response in the thin, flexible lamella, resulting in its bending to accommodate the gradients. Our ultrafast 4D STEM approach enables virtual imaging through postselection of masks in diffraction space (Figure 1c) while also providing quantitative strain measurements through analysis of the diffraction patterns (Figure 1d).^36^ The determination and calibration of the temporal overlap between the optical pump and electron probe (time zero) are described in detail in the SI.

**Figure 1:**
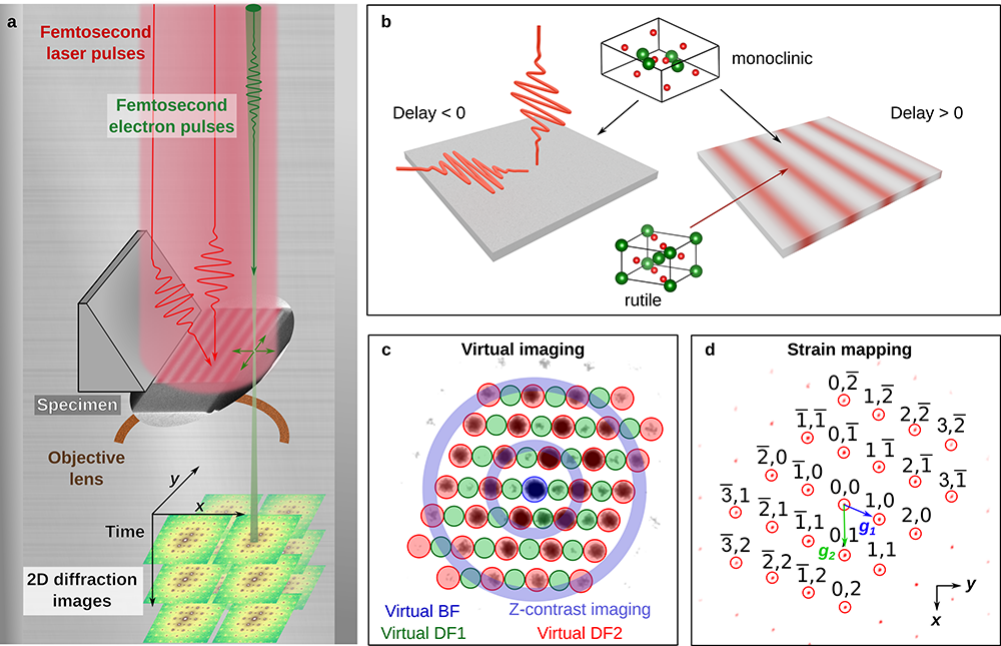
Ultrafast 4D STEM experiments. (a) Schematic of the experimental setup: femtosecond laser pulses induce a patterned structural phase transition in a vanadium dioxide lamella, which is locally probed in real space using delayed femtosecond electron pulses. (b) A transient laser grating triggers a structural phase transition, resulting in different lattice structures in separate domains of the same lamella. (c) Arbitrary virtual masks enable the generation of ultrafast virtual images. (d) Ultrafast strain mapping captures the dynamics of the structural phase transition by measuring the distance between multiple diffraction spots as a function of position on the specimen.

## Results and Discussion

### Photodriven Ultrafast Structural Phase Transition

The diffraction patterns corresponding to the monoclinic (M1) and rutile (R) phases are shown in Figure 2a. The M1 superstructure is characteristic of the monoclinic phase and is absent in the rutile phase, whereas the stronger diffraction spots are common to both phases. Ultrafast bright-field imaging of the VO_2_ lamella shows the laser-induced grating (Figure 2b). Variations in the bright-field intensity between neighboring regions arise from diffraction-contrast effects. Small local changes in specimen tilt or bending can shift regions slightly in or out of the Bragg condition, redistributing diffracted intensity among the Bragg reflections and the transmitted beam. As a result, BF intensity can vary locally even though the material thickness remains unchanged. The fringes are predominantly straight, reflecting the optical interference geometry, with intensity modulations along the grating lines. While the real-space bright-field contrast visualizes the spatial modulation, it does not provide direct quantitative information on the structural phase state. To overcome this limitation, we perform additional 4D STEM on the indicated window in Figure 2b and extract quantitative information (Figure 2c). This allows us to track the structural phase transition in space and time and quantify phase coexistence by diffraction analysis. In regions close to maximum interference of the optical grating, we observe a drop of the M1-phase–exclusive Bragg spots by 75%, while in other regions, the monoclinic phase appears slightly enhanced. The drop in intensity of the M1 spots reflects the local progression of the structural phase transition from the monoclinic to the rutile phase. Intensity variations exceeding the normalized value of 1 (red line in Figure 2c) reflect relative increases compared to the prepump state. Such increases arise from local sample bending and tilts, which can shift local regions closer to the Bragg condition, modifying the intersection of the reciprocal lattice with the Ewald sphere and thereby enhancing the diffracted intensity. Figures 2d,e show line profiles and corresponding analysis of the grating peak width as a function of the pump–probe delay. While bright-field contrast does not provide a quantitative measure of either strain or phase fraction, it serves as a sensitive real-space indicator of the overall photoinduced response. In particular, the evolution of the grating profile allows us to qualitatively follow the spatiotemporal redistribution of contrast on ultrafast time scales. We note that the spatial position of the third grating maximum shifts as a function of pump–probe delay (Figure 2d), likely reflecting transient lattice distortions induced by photoexcited acoustic waves, which can locally modify specimen tilt and thereby alter the bright-field contrast distribution. From the analysis of the time-dependent grating line profiles, we identify two characteristic time scales (Figure 2e). The first time constant, approximately 2 ps, is consistent with the time scale of the photoinduced structural phase transition reported in previous ultrafast diffraction studies.^34^ The absorbed fluence at the interference maxima locally exceeds the reported threshold for the photoinduced insulator–metal transition in VO_2_, confirming that the excitation conditions are sufficient to trigger the structural phase transition. We note that the observed ∼2 ps time scale is comparable to the electron pulse duration (∼1.5 ps) and therefore represents the effective temporal resolution of our measurements; the intrinsic transition occurs on faster, femtosecond time scales. The second, slower time scale of about 130 ps reflects the gradual build-up and real-space evolution of the grating contrast following excitation. The characteristic time constants are convoluted with the electron beam pulse duration, which is approximately 1.5 ps, as determined through photon-induced near-field electron microscopy. Importantly, the bright-field contrast does not directly track the evolution of the structural phase transition. The bright-field contrast reflects a convolution of multiple contributions, including diffuse scattering, transient lattice disorder, and local bending or tilts. These effects respond differently to optical excitation and are not phase-locked to the structural order parameter. Consequently, while bright-field imaging captures the overall spatial response to photoexcitation, the direct identification and tracking of the phase transition requires Bragg-resolved diffraction and quantitative strain analysis, as provided by ultrafast 4D STEM.

**Figure 2:**
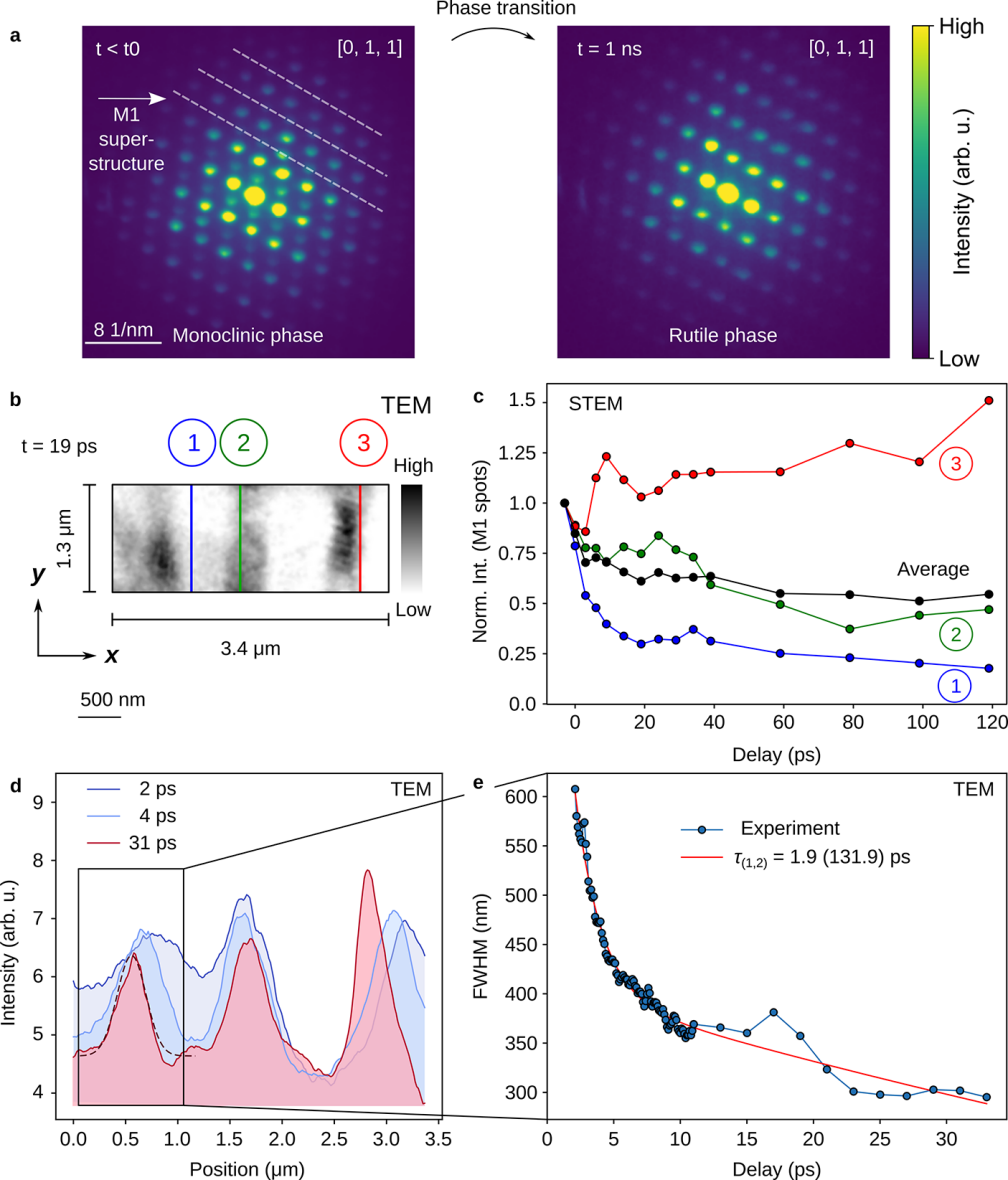
Transient grating excitation in vanadium dioxide on ultrafast time scales. (a) Ultrafast electron diffraction patterns before and after the structural phase transition. The weak diffraction spots corresponding to the M1 superstructure, which appear at positions in between the main rutile reflections (effectively on every second line of the diffraction pattern), disappear upon completion of the transition to the rutile phase at 1 ns. (b) Ultrafast bright-field imaging following patterned light excitation. (c) Normalized intensity of the M1-exclusive spots, integrated over the line profiles of 4D diffraction scans performed in the highlighted window in (b). (d) Line profiles of the ultrafast bright-field images in (b) at different time-delays. (e) FWHM of Gaussian fits to the line profiles in (d) as a function of the pump–probe delay.

### Ultrafast Virtual Imaging

We employ the unique capabilities of ultrafast 4D STEM and apply several virtual dark-field masks that give us complementing virtual imaging capabilities. The first panel of Figure 3a (top) illustrates the structural phase transition by including the M1-exclusive Bragg spots through virtual imaging. As the material transitions from the M1 to the R phase, the intensity of these M1-exclusive spots is strongly suppressed (Figure 2c), resulting in a corresponding decrease in the intensity of the virtual images at regions of high local fluence. Although the M1-exclusive reflections provide a direct fingerprint of the monoclinic phase, their diffraction intensities are significantly weaker than those of the shared reflections. Under strong photoexcitation, transient strain and diffraction condition changes further reduce their contrast; therefore, the spatial modulation is often more clearly visible in the shared Bragg reflections. Panels 2, 3, and 4 of Figure 3a show complementary information by unmasking the shared spots of the monoclinic and rutile phase and applying different annular dark-field rings. While the various STEM contrasts exhibit modulation following the periodicity of the standing light field, strong diagonal features appear across the lamella. These primarily arise from static bending and local diffraction condition changes in the FIB-prepared VO_2_ lamella (Figure S1), rather than from optical illumination. Thickness variations are minor, and under photoexcitation, transient acoustic waves further modulate diffraction and bright field contrast. Despite these static and dynamic effects, the known grating periodicity and orientation allow excitation-driven responses to be distinguished from sample-related features. Recent studies suggest that virtual dark-field imaging may be directly sensitive to strain fields.^37^

**Figure 3:**
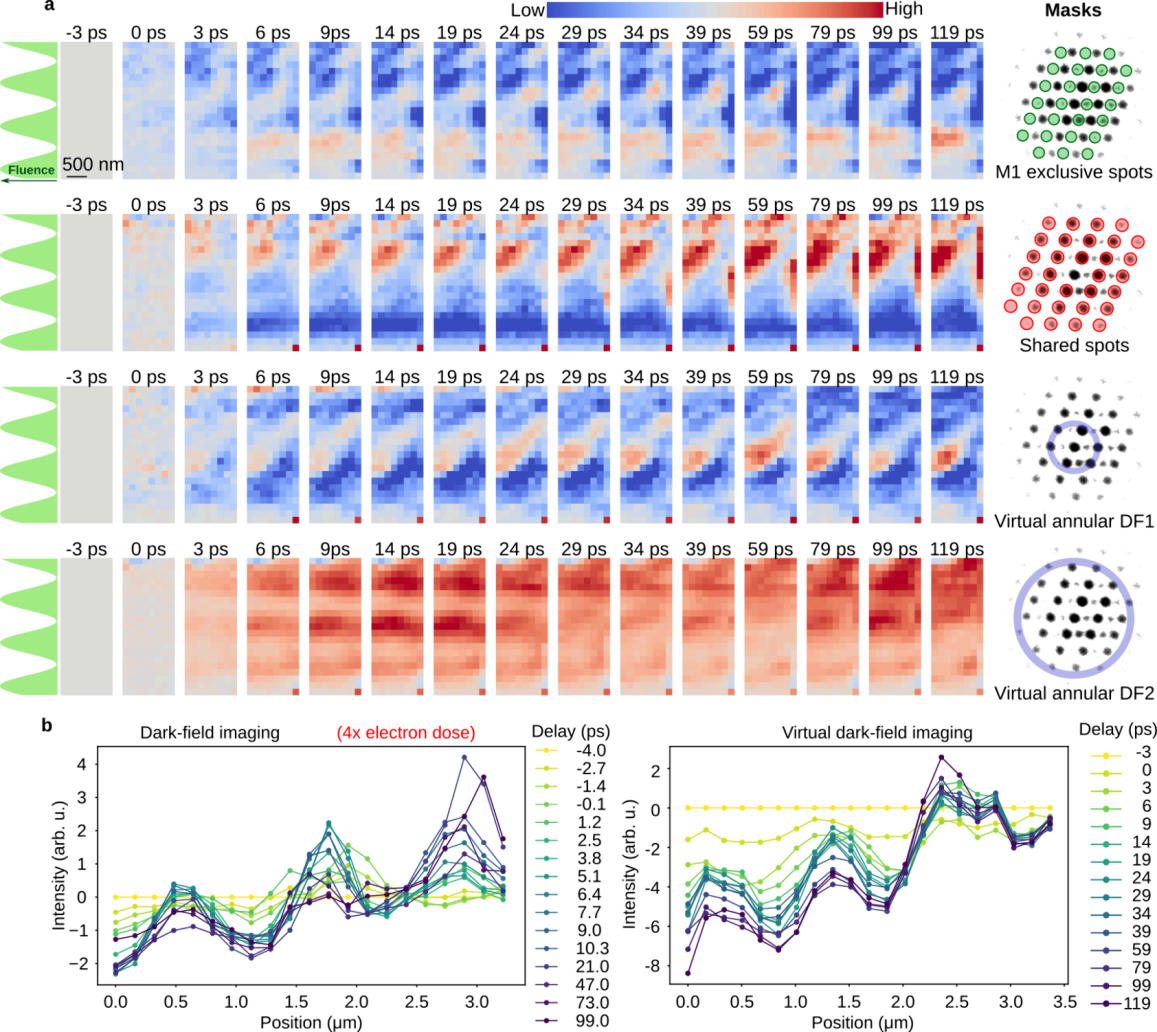
Dark-field imaging of a photodriven phase transition. (a) Ultrafast diffraction-contrast imaging using various virtual masks (right panel) applied to the region shown in Figure 2b. Different virtual masks highlight distinct specimen properties. For instance, specific masks can enhance the contrast of weak reflections or provide varying Z-contrast by selecting different scattering angles. The grating direction and spacing are indicated in green on the left side. (b) Comparison of line profiles from dark-field imaging using a single physical aperture (5 μm diameter) covering a single M1-exclusive spot versus virtual dark-field imaging incorporating all M1-exclusive spots, highlighting comparable contrast achieved in the latter with reduced electron dose.

Figure 3b shows that 4D STEM virtual dark-field imaging provides a similar contrast to conventional dark-field TEM, where a physical aperture selects a single diffraction spot. In virtual imaging, multiple diffraction spots can be selected simultaneously through postprocessing rather than a single mechanical aperture, resulting in a higher signal-to-noise ratio (SNR) at a lower effective electron dose. For the comparison shown here, the STEM dwell time was adjusted such that the total electron dose over a given field of view was on the same order of magnitude as in the corresponding TEM dark-field measurements. The TEM images were additionally downsampled to match the effective spatial resolution of the STEM data, and comparable fields of view were selected. Under these conditions, the comparison emphasizes the qualitative robustness of virtual dark-field imaging rather than serving as a quantitative performance benchmark, which is beyond the scope of this work.

The SNR can be further improved during postanalysis, for example, by subtracting diffuse scattering contributions. Virtual apertures also eliminate the need for complex, sample-specific dark-field aperture arrays designed to collect multiple diffraction spots simultaneously, as employed in ref 38. While the overall contrast trends remain similar, conventional bright- or dark-field imaging is sensitive to local sample bending and tilts, which can shift regions of the specimen out of the selected diffraction condition. In contrast, virtual dark-field imaging in 4D STEM sums over all equivalent diffraction spots, making it largely robust against such distortions. Achieving similar contrast at reduced electron dose is particularly important for time-resolved measurements, where repeated pump–probe cycles can otherwise lead to cumulative beam damage. By extracting more information from each electron interacting with the specimen, ultrafast 4D STEM reduces the total number of electrons required for imaging, thereby minimizing both electron- and laser-induced damage.

### Correlative Ultrafast Imaging

Further, we compare bright-field imaging (Figure 4a) with strain mapping (Figure 4b) derived from 4D STEM. Strain imaging quantifies atomic displacements in reciprocal space, whereas bright-field imaging captures intensity variations in the direct (nondiffracted) beam arising from local changes in diffraction conditions, which can produce complex signals. In both cases, the laser-induced grating is clearly visible (Figure 4c). Bending effects, resulting from strain gradients, appear weaker near the sample edge, where the lamella remains attached to the bulk, likely due to mechanical constraints suppressing out-of-plane deformation. In contrast, the in-plane strain components remain relatively uniform across the lamella. All images are normalized to negative time delays to account for static bending and strain conditions present prior to excitation. Regions where strain retrieval fails (black crosses, Figure 4b) correspond to locations where fitting of the diffraction peaks was unsuccessful due to a low signal or local deviations from ideal diffraction conditions. Other strain components are discussed in the SI.

**Figure 4:**
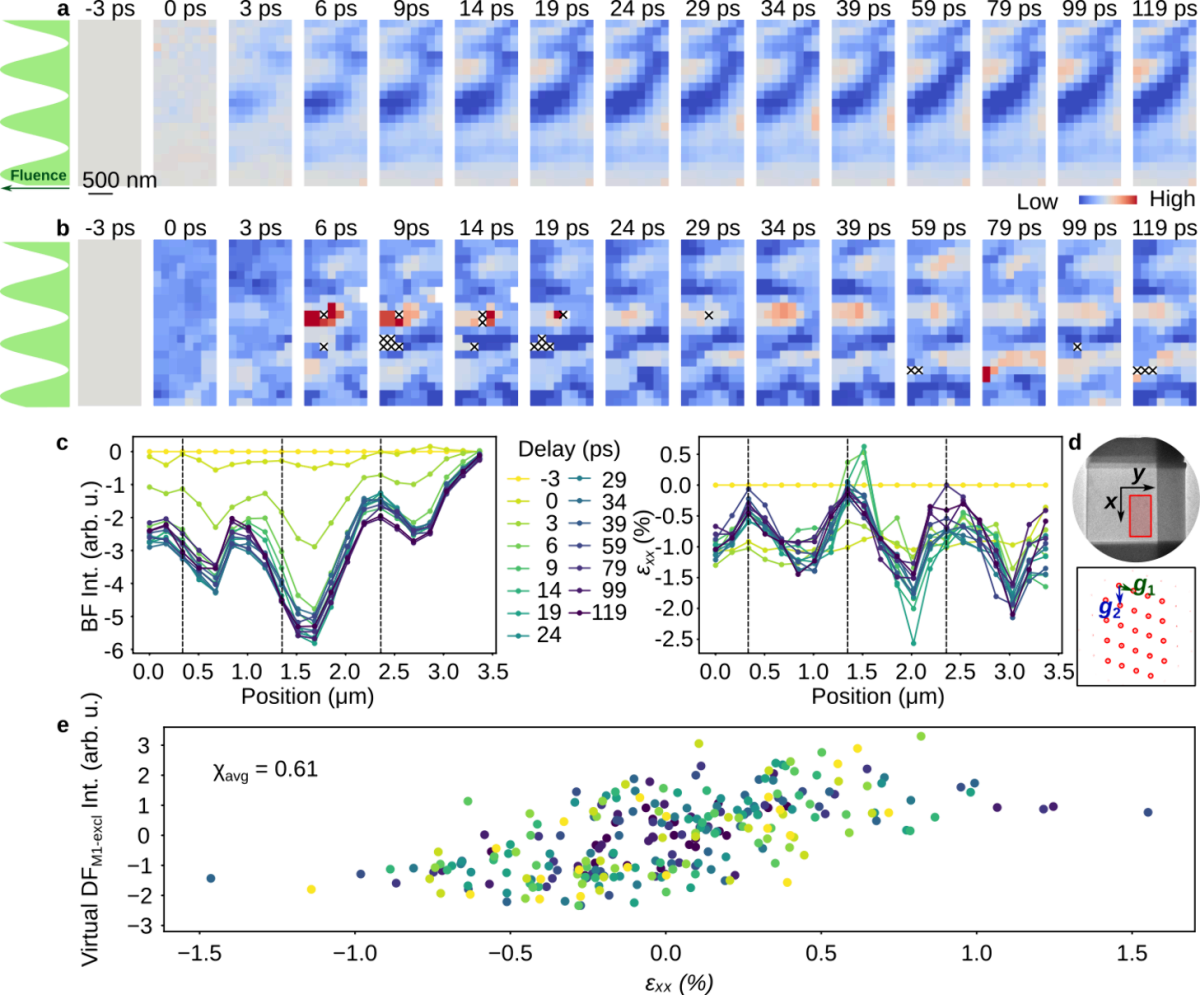
Bright-field imaging and strain analysis following transient grating excitation. (a) Ultrafast bright-field imaging, showing contrast contributions from bending, diffraction, and other scattering effects. (b) Strain mapping from local diffraction pattern analysis with black crosses indicating positions where strain retrieval was unsuccessful. The grating direction and spacing is indicated in green on the left side. (c) Integrated line profiles from virtual bright-field imaging, highlighting contributions from bending and diffraction contrast. The profiles are integrated along the *y*-direction. In both measurements, grating oscillations are clearly visible. (d) Measurement orientation in real and diffraction space. (e) Correlation plot of detrended virtual dark-field imaging (Figure 3a panel 1) and strain indicating a positive correlation.

To directly relate the observed lattice strain to the structural phase transition, we next quantify the correlation between the strain maps and Bragg-resolved virtual dark-field imaging sensitive to the monoclinic M1 phase (Figure 4e). Following laser excitation, we observed a correlation coefficient of approximately 0.6 between the M1-specific dark-field signal and the strain maps, indicating a strong positive correlation. The strain vector in our system is directly related to changes in the lattice parameter *a*, which contracts by approximately 1% during the phase transition.^39^ This confirms that the measured strain arises as a direct consequence of the structural-phase transition. These findings underscore the value of quantitatively resolving strain evolution, providing clearer insights into the temporal and spatial coupling between structural transformations and the lattice strain. Interestingly, strain and virtual bright-field imaging show no direct correlation; this is discussed in more detail in the SI.

To further support our conclusions, we performed finite element simulations (see SI for more information) in which a VO_2_ lamella is heated by a transient laser grating without including contributions from the phase transition. These simulations show that, even at elevated temperatures, the resulting strain is insufficient to significantly shift the metal-to-insulator transition^15^ or to trigger the phase transition. The maximum simulated strain is roughly 1 order of magnitude smaller than the experimentally observed strain, indicating that thermal effects alone cannot account for the measured lattice deformation. In terms of time scale, the simulated strain field builds up over approximately 50 ps, whereas the experimental strain reaches its maximum after about 20 ps and remains constant thereafter. This faster rise reflects the combination of the ultrafast structural phase transition, which occurs on subpicosecond time scales, and the subsequent mechanical redistribution of lattice deformation within the lamella. The differences in both amplitude and temporal evolution therefore demonstrate that the observed strain predominantly originates from the structural phase transition rather than from laser-induced heating. Furthermore, the experimentally determined strain, on the order of 1%, is too small to appreciably affect the phase transition temperature, as it is expected to shift the transition temperature by less than ∼10 K,^15^ which is negligible under our experimental conditions.

## Conclusions

In this work, we demonstrated the simultaneous mapping of strain and ultrafast imaging to capture a photoinduced phase transition in a vanadium dioxide lamella. While strain does not initiate the transition in our case, it can play a critical role in modulating the transformation by locally lowering the energy barrier and influencing the transition temperature.^40^ By resolving quantitative strain dynamics with nanometer spatial and picosecond temporal resolution, we reveal how structural distortions emerge as a consequence of the phase transition. These insights enable rational strain-engineering strategies, such as controlling phase nucleation sites, tuning transition thresholds, and stabilizing metastable phases through designed strain fields, which are critical for the development of ultrafast electronic components, phase-change materials, and next-generation energy-efficient switching devices.

We further demonstrated the creation of a transient transmission grating on picosecond time scales driven by the photoinduced phase transition and the accompanying refractive-index modulation. Through band gap engineering, via strain, doping, or compositional tuning, this mechanism can in principle be shifted into the telecom wavelength range (1550 nm), where low-loss optical-fiber communication operates. In this regime, the optically written transient gratings could function as ultrafast, reconfigurable diffraction elements, enabling spectral filtering, optical switching, and dynamic beam steering. More broadly, this approach establishes a materials-centric route to integrated photonic devices in which light propagation is actively controlled through phase transition-driven structural and optical responses.

Additionally, we showed that virtual 4D STEM imaging provides phase-selective diffraction contrast comparable to conventional dark-field TEM while simultaneously enabling quantitative strain mapping from the same data set. The reduced effective electron dose is particularly important for time-resolved measurements, where many pump–probe repetitions are required and cumulative beam damage can otherwise limit achievable data quality. Local bending and tilt variations induced by ultrafast thermoelastic stress are intrinsic to strongly excited thin lamellae and can influence diffraction contrast. Future implementations incorporating ultrafast precession 4D STEM, optimized sample geometries, or STEM-EELS to directly probe phase-specific electronic and chemical signatures could further reduce diffraction-condition effects and enable even clearer isolation of structural phase transitions.

Looking ahead, we envision 4D STEM as a key technique in ultrafast transmission electron microscopy for probing structural and electronic dynamics during photoinduced phase transitions. While its first applications are still emerging, we anticipate significant advancements adapted from static 4D STEM. Improvements in direct-electron detectors and hardware synchronization will push the technique toward atomic resolution, as this regime requires not only single-electron sensitivity but also large dynamic range, and high detection efficiency. Further developments, such as electron ptychography and center-of-mass analysis, could enable full ultrafast phase imaging, including direct visualization of transient charge redistribution and other electronic degrees of freedom—an achievement that remains highly challenging with current off-axis electron holography on ultrafast time scales.^41^ Future progress will depend on the development of more coherent pulsed electron sources with higher electron flux, improved direct-electron detectors, and enhanced mechanical stability. These advancements will continue to expand the capabilities of ultrafast 4D STEM, opening new possibilities for high-speed, high-resolution imaging in condensed matter physics and materials science.

## Methods

### Sample Preparation

Single crystals of VO_2_ were grown by isothermal flux evaporation from 99.99% V_2_O_5_ powder (Sigma-Aldrich) in a flowing nitrogen atmosphere at 1000 °C, following established procedures.^42,43^ Thin lamellas were prepared from VO_2_ needles using focused ion beam (FIB) milling, yielding specimens with lateral dimensions of approximately 4 × 5 μm^2^ and a thickness of ∼150 nm. The lamellas were mounted on a Gatan double-tilt holder for transmission electron microscopy experiments. Sample thickness was independently verified using electron energy-loss spectroscopy (EELS).

### Ultrafast Electron Microscopy and Pump–Probe Configuration

Ultrafast electron diffraction and 4D STEM experiments were performed at the Ultrafast Electron Microscopy (UEM) laboratory at the KTH Royal Institute of Technology (Stockholm, Sweden). The experiments were conducted at room temperature in a pump–probe configuration. Photoelectron probe pulses with a full width at half-maximum (FWHM) duration of approximately 1.5 ps were generated and temporally characterized using photon-induced near-field electron microscopy (PINEM).

The optical pump pulses were derived from the same laser source (Tangerine, Amplitude Systemes) with a photon energy of ∼1.2 eV and a pulse duration of 300 fs (FWHM). The pump beam was focused to a spot size of approximately 200 μm on the sample. A 35° slanted aluminum-coated mirror with a reflectivity of around 50% was positioned above the sample plane to generate a transient optical grating via interference between the direct pump beam and its reflection. This configuration produced a sinusoidal excitation pattern with a spatial periodicity of 1 μm.

The relative time delay between the pump and probe pulses was controlled by using a motorized delay stage (Newport ESP301). Experiments were performed at a repetition rate of 12 kHz, ensuring the complete relaxation of the sample between successive excitation cycles.

### Ultrafast 4D STEM Acquisition

In ultrafast 4D STEM measurements, a focused electron probe was rastered across the sample, and a full diffraction pattern was recorded at each real-space position and pump–probe delay. This approach enables simultaneous acquisition of spatially resolved diffraction data and virtual imaging modes. Diffraction patterns were recorded using a hybrid pixel detector (CheeTah T3, Amsterdam Scientific Instruments), providing high sensitivity and dynamic range.

For 4D STEM scans, a condenser aperture with a diameter of 50 μm was used to generate a quasi-parallel electron beam with a convergence semiangle of a few milliradians. This configuration enabled clear separation of individual diffraction peaks, facilitating Bragg-resolved virtual imaging and quantitative strain analysis. The electron beam brightness was optimized to balance diffraction-space resolution and real-space imaging performance while minimizing electron dose.

## Supplementary Material



## Notes

Sample preparation, COMSOL simulations of strain from laser-induced heating, ultrafast transmission electron microscopy, ultrafast 4D STEM data analysis, ultrafast strain mapping, reproducibility of strain mapping, and time0 determination (PDF)

## References

[1] Mitrano M., Cantaluppi A., Nicoletti D., Kaiser S., Perucchi A., Lupi S., Di Pietro P., Pontiroli D., Riccò M., Clark S. R., Jaksch D., Cavalleri A. (2016). Possible light-induced
superconductivity in K_3_C_60_ at high temperature. Nature.

[2] Samizadeh
Nikoo M., Soleimanzadeh R., Krammer A., Migliato
Marega G., Park Y., Son J., Schueler A., Kis A., Moll P. J. W., Matioli E. (2022). Electrical control of glass-like
dynamics in vanadium dioxide for data storage and processing. Nat. Electron.

[3] Ohkoshi S.-i., Tsunobuchi Y., Matsuda T., Hashimoto K., Namai A., Hakoe F., Tokoro H. (2010). Synthesis of a metal
oxide with a room-temperature photoreversible phase transition. Nature Chem..

[4] Yang Z., Ko C., Ramanathan S. (2011). Oxide Electronics Utilizing Ultrafast Metal-Insulator
Transitions. Annu. Rev. Mater. Res..

[5] Gu Q., Falk A., Wu J., Ouyang L., Park H. (2007). Current-Driven
Phase Oscillation and Domain-Wall Propagation in W_x_V_1‑x_O_2_ Nanobeams. Nano
Lett..

[6] Kim B.-J., Lee Y. W., Chae B.-G., Yun S. J., Oh S.-Y., Kim H.-T., Lim Y.-S. (2007). Temperature
dependence of the first-order
metal-insulator transition in VO_2_ and programmable critical
temperature sensor. Appl. Phys. Lett..

[7] Dicken M. J., Aydin K., Pryce I. M., Sweatlock L. A., Boyd E. M., Walavalkar S., Ma J., Atwater H. A. (2009). Frequency
tunable near-infrared metamaterials based on VO_2_ phase
transition. Opt. Express.

[8] Driscoll T., Kim H.-T., Chae B.-G., Di Ventra M., Basov D. N. (2009). Phase-transition driven memristive system. Appl. Phys. Lett..

[9] Ruzmetov D., Gopalakrishnan G., Deng J., Narayanamurti V., Ramanathan S. (2009). Electrical
triggering of metal-insulator transition
in nanoscale vanadium oxide junctions. J. Appl.
Phys..

[10] Strelcov E., Lilach Y., Kolmakov A. (2009). Gas Sensor
Based on MetalInsulator
Transition in VO_2_ Nanowire Thermistor. Nano Lett..

[11] Briggs R.
M., Pryce I. M., Atwater H. A. (2010). Compact silicon photonic waveguide
modulator based on the vanadium dioxide metal-insulator phase transition. Opt. Express.

[12] Ruzmetov D., Gopalakrishnan G., Ko C., Narayanamurti V., Ramanathan S. (2010). Three-terminal
field effect devices utilizing thin
film vanadium oxide as the channel layer. J.
Appl. Phys..

[13] Sood A., Shen X., Shi Y., Kumar S., Park S. J., Zajac M., Sun Y., Chen L.-Q., Ramanathan S., Wang X., Chueh W. C., Lindenberg A. M. (2021). Universal
phase dynamics in VO_2_ switches revealed by ultrafast operando
diffraction. Science.

[14] Bertoni R., Lorenc M., Cailleau H., Tissot A., Laisney J., Boillot M.-L., Stoleriu L., Stancu A., Enachescu C., Collet E. (2016). Elastically driven
cooperative response of a molecular
material impacted by a laser pulse. Nat. Mater..

[15] Park J. H., Coy J. M., Kasirga T. S., Huang C., Fei Z., Hunter S., Cobden D. H. (2013). Measurement
of a solid-state triple
point at the metal–insulator transition in VO_2_. Nature.

[16] Johnson A. S. (2022). Ultrafast X-ray imaging of the light-induced phase transition in
VO_2_. Nat. Phys..

[17] O’Callahan B.
T., Jones A. C., Hyung Park J., Cobden D. H., Atkin J. M., Raschke M. B. (2015). Inhomogeneity
of the ultrafast insulator-to-metal transition
dynamics of VO_2_. Nat. Commun..

[18] McKenna A. J., Eliason J. K., Flannigan D. J. (2017). Spatiotemporal
Evolution of Coherent
Elastic Strain Waves in a Single MoS_2_ Flake. Nano Lett..

[19] Zhang Y., Flannigan D. J. (2019). Observation of Anisotropic Strain-Wave
Dynamics and
Few-Layer Dephasing in MoS_2_ with Ultrafast Electron Microscopy. Nano Lett..

[20] Du D. X., Flannigan D. J. (2020). Imaging phonon dynamics with ultrafast
electron microscopy:
Kinematical and dynamical simulations. Structural
Dynamics.

[21] Zhang Y., Flannigan D. J. (2021). Imaging
Nanometer Phonon Softening at Crystal Surface
Steps with 4D Ultrafast Electron Microscopy. Nano Lett..

[22] Ji S., Grånäs O., Kumar Prasad A., Weissenrieder J. (2022). Influence
of strain on an ultrafast phase transition. Nanoscale.

[23] Wu J., Prasad A. K., Balatsky A., Weissenrieder J. (2024). Spatiotemporal
determination of photoinduced strain in a Weyl semimetal. Structural Dynamics.

[24] Huber M. A., Plankl M., Eisele M., Marvel R. E., Sandner F., Korn T., Schüller C., Haglund R. F. J., Huber R., Cocker T. L. (2016). Ultrafast Mid-Infrared
Nanoscopy of Strained Vanadium
Dioxide Nanobeams. Nano Lett..

[25] Dönges S. A., Khatib O., O’Callahan B.
T., Atkin J. M., Park J. H., Cobden D., Raschke M. B. (2016). Ultrafast Nanoimaging
of the Photoinduced Phase Transition Dynamics in VO_2_. Nano Lett..

[26] Zewail A. H. (2006). 4D Ultrafast
Electron Diffraction, Crystallography, and Microscopy. Annu. Rev. Phys. Chem..

[27] Kim Y.-J., Park W.-W., Nho H.-W., Kwon O.-H. (2024). High-resolution
correlative imaging in ultrafast electron microscopy. Advances in Physics: X.

[28] Feist A., Rubiano Da Silva N., Liang W., Ropers C., Schäfer S. (2018). Nanoscale
diffractive probing of strain dynamics in ultrafast transmission electron
microscopy. Structural Dynamics.

[29] Nakamura A., Shimojima T., Ishizaka K. (2022). Visualizing optically-induced strains
by five-dimensional ultrafast electron microscopy. Faraday Discuss..

[30] Shimojima T., Nakamura A., Ishizaka K. (2023). Development of five-dimensional scanning
transmission electron microscopy. Rev. Sci.
Instrum..

[31] Rozeveld S. J., Howe J. M. (1993). Determination of
multiple lattice parameters from convergent-beam
electron diffraction patterns. Ultramicroscopy.

[32] Ophus C. (2019). Four-Dimensional
Scanning Transmission Electron Microscopy (4D-STEM): From Scanning
Nanodiffraction to Ptychography and Beyond. Microsc. Microanal..

[33] Cao G., Jiang S., Åkerman J., Weissenrieder J. (2021). Femtosecond
laser driven precessing magnetic gratings. Nanoscale.

[34] Baum P., Yang D.-S., Zewail A. H. (2007). 4D Visualization
of Transitional
Structures in Phase Transformations by Electron Diffraction. Science.

[35] Lobastov V. A., Weissenrieder J., Tang J., Zewail A. H. (2007). Ultrafast Electron
Microscopy (UEM): Four-Dimensional Imaging and Diffraction of Nanostructures
during Phase Transitions. Nano Lett..

[36] Savitzky B. H. (2021). py4DSTEM: A Software
Package for Four-Dimensional Scanning Transmission
Electron Microscopy Data Analysis. Microsc.
Microanal..

[37] Gammer C., Burak Ozdol V., Liebscher C. H., Minor A. M. (2015). Diffraction contrast
imaging using virtual apertures. Ultramicroscopy.

[38] Danz T., Domröse T., Ropers C. (2021). Ultrafast nanoimaging of the order
parameter in a structural phase transition. Science.

[39] Kucharczyk D., Niklewski T. (1979). Accurate X-ray determination of the lattice parameters
and the thermal expansion coefficients of VO_2_ near the
transition temperature. J. Appl. Crystallogr..

[40] Aetukuri N. B., Gray A. X., Drouard M., Cossale M., Gao L., Reid A. H., Kukreja R., Ohldag H., Jenkins C. A., Arenholz E., Roche K. P., Dürr H. A., Samant M. G., Parkin S. S. P. (2013). Control of the
metal–insulator
transition in vanadium dioxide by modifying orbital occupancy. Nature Phys..

[41] Houdellier F., Caruso G. M., Weber S., Hÿtch M. J., Gatel C., Arbouet A. (2019). Optimization of off-axis electron
holography performed with femtosecond electron pulses. Ultramicroscopy.

[42] Sasaki H., Watanabe A. (1964). A New Growing Method
for VO_2_ Single Crystals. J. Phys.
Soc. Jpn..

[43] Aramaki S., Roy R. (1968). Single-crystal growth of VO_2_ by isothermal flux-evaporation. J. Mater. Sci..

