# Supplementary material for: Correlative Ultrafast Imaging of a Photodriven Phase Transition Using 4D Scanning Transmission Electron Microscopy
Source: ACS Nano. 2026 Jun 8;20(24):17264–72. doi: 10.1021/acsnano.5c22662 (PMC13296485; doi:10.1021/acsnano.5c22662)
Supplement: Supplementary file 1 [file nn5c22662_si_001.pdf]

Correlative Ultrafast Imaging of a Photo-Driven Phase Transition Using 4D Scanning Transmission Electron Microscopy

Arthur Niedermayr ,*,† Jianyu Wu ,† Bertina Fisher ,‡ Ido Kaminer ,¶ and Jonas Weissenrieder *,†

†*Department of Materials and Nano Physics, School of Engineering Sciences, KTH Royal Institute of Technology, Stockholm SE-100 44, Sweden.*

‡*Department of Physics, Technion–Israel Institute of Technology, Haifa 32000, Israel.*

¶*Department of Electrical and Computer Engineering, Technion–Israel Institute of Technology, Haifa 32000, Israel.*

E-mail: arthurn@kth.se; jonas@kth.se

Supporting Information

Correlative Ultrafast Imaging of a Photo-Driven Phase Transition Using 4D Scanning Transmission Electron Microscopy

Arthur Niedermayr,[1,*] Jianyu Wu,[1] Bertina Fisher,[2] Ido Kaminer,[3] and Jonas Weissenrieder[1,*]

[1]Department of Materials and Nano Physics, School of Engineering Sciences, KTH Royal Institute of Technology, SE-100 44 Stockholm, Sweden

[2]Department of Physics, Technion–Israel Institute of Technology, Haifa 32000, Israel

[3]Department of Electrical and Computer Engineering, Technion–Israel Institute of Technology, Haifa 32000, Israel

* E-mail: arthurn@kth.se, jonas@kth.se

Sample preparation

Single crystals of VO_2, have been grown by isothermal flux evaporation[1,2] from 99.99% V_2O_5 powder (Sigma Aldrich), in an atmosphere of flowing nitrogen at 1000°C. A single as-grown VO_2, needle, free of cracks and voids, was chosen for further processing with a dimension of about $0.05 \times 0.05 \times 3\,\text{mm}^3$.

Focused ion beam (FIB) milling was performed on this VO_2 needle to create a FIB lamella with dimensions of $4 \times 5\,\mu\text{m}^2$ and a thickness of approximately 150 nm (Fig. S1). FIB milling was carried out using a Ga ion source operated at 30 kV. Final thinning was performed at low ion energies to minimize Ga implantation and surface damage.

The strain measurements shown in Figs. 2-4 are obtained from a $1.3\,\mu\text{m} \times 3.4\,\mu\text{m}$ region (Fig. S1) selected for its low defect density and clear diffraction features. Similar measurements repeated on a separate region of the sample yield qualitatively consistent spatial strain

profiles, confirming reproducibility, although these additional datasets are not shown here for clarity.

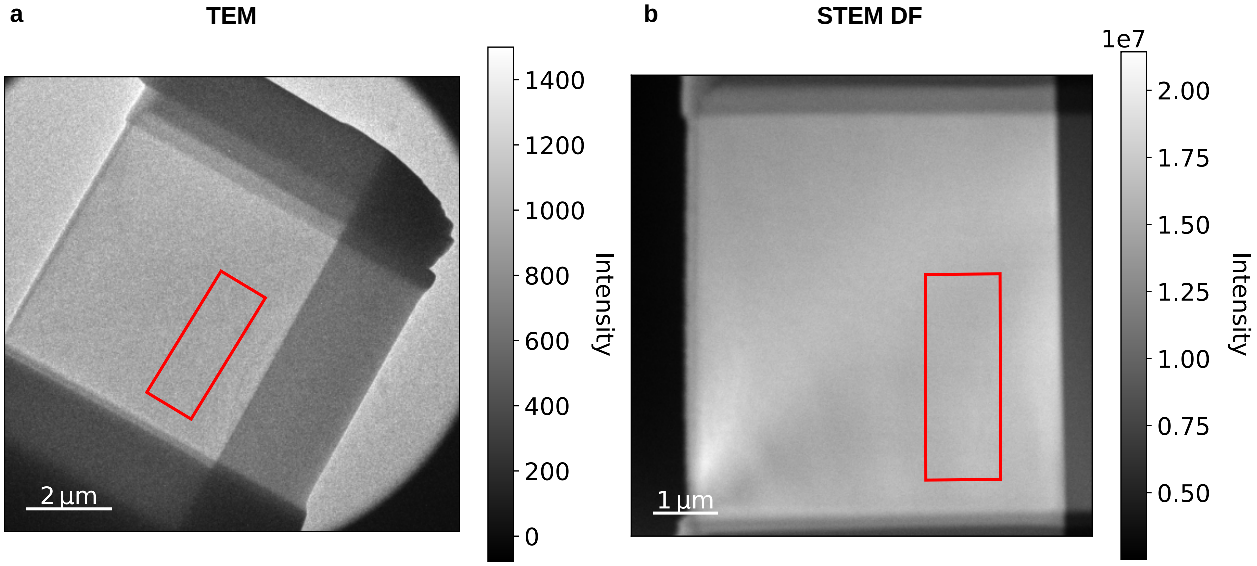

Figure S1: **VO_2 lamella.** (a) TEM, (b) STEM DF. The red rectangle indicates the area where ultrafast STEM measurements were performed.

COMSOL simulations of strain from laser-induced heating

To rule out strain as the primary driver of the structural phase transition, we performed finite element simulations (using the COMSOL software package) of laser-induced heating in the monoclinic phase of a VO_2 lamella. Even under exaggerated excitation conditions—approximately twice the experimental laser fluence and with no phase transition explicitly modeled—the resulting strain reaches only 0.1% after 50 ps, an order of magnitude smaller than the strain observed in the experiment. In these simulations, the VO_2 lamella reaches a maximum temperature of around 500 K (Fig. S2a), which exceeds the bulk phase transition temperature of VO_2 at $\sim$340 K. The associated laser profile is shown in Fig. S2b, and the resulting strain evolution confirms that the heating-induced strain remains negligible. If a lower pump intensity were used, the maximum temperature and the resulting strain would be reduced further. These results therefore provide a conservative, upper-limit estimate, indicating that the observed strain is not caused by laser-induced heat-

ing and is highly unlikely to trigger the phase transition. Moreover, literature reports suggest that significantly higher strain values would be required to meaningfully shift the transition temperature in vanadium dioxide.[3]

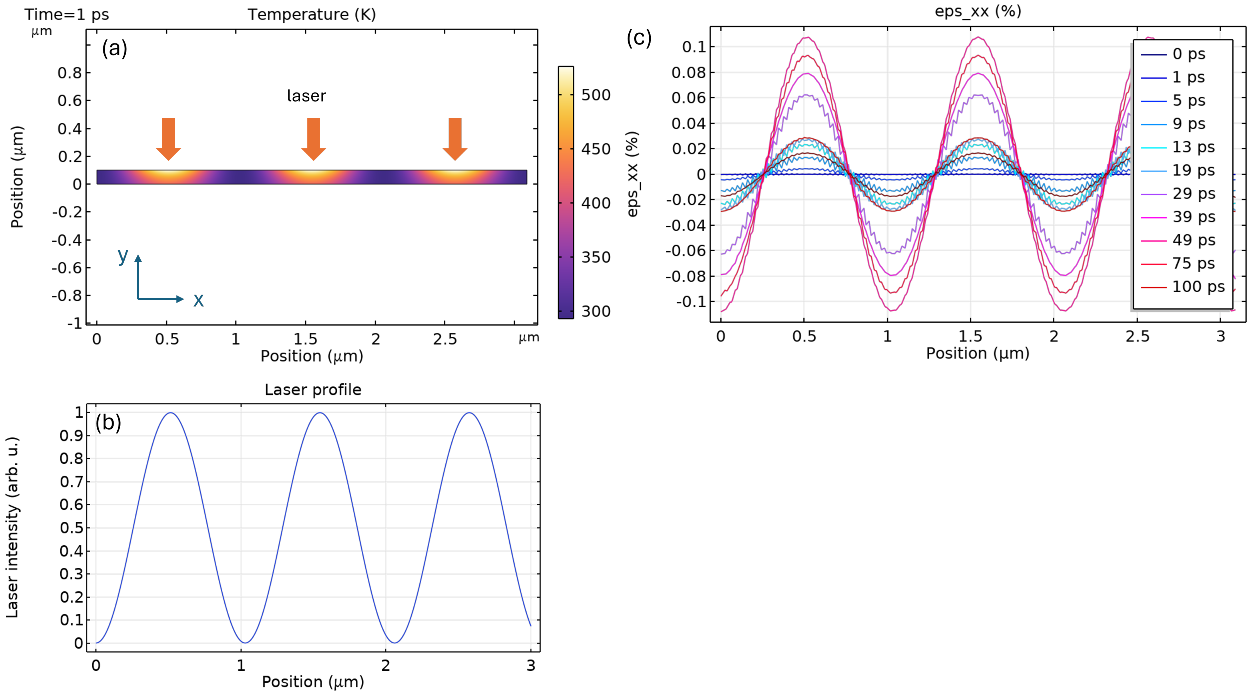

Figure S2: **COMSOL simulations.** (a) Temperature distribution of a vanadium dioxide lamella heated by a transient laser grating. (b) Laser intensity profile used in (a) to heat the sample. (c) Simulated strain component ε_{xx} resulting from laser-induced heating. No phase transition was included in this model, so it does not contribute to the strain. The simulated strain values remain an order of magnitude smaller than those observed experimentally.

The pump laser was modeled as a 300 fs pulse applied at $t = 0$ ps, with a volumetric power of 2.2×10^{21} W/m^3. The laser power along the x-axis was modulated sinusoidally to simulate a transient optical grating (TOG). Light absorption in the thickness direction was modeled using the Beer-Lambert law. The sample was treated as free-standing, with periodic boundary conditions applied to the left and right sides. VO_2 material properties were taken from the COMSOL material library.

The following parameters were used for the COMSOL simulations:

- Sample thickness: 100 nm

- Absorption coefficient: 5.9429×10^4 cm^{-1} [4]
- Heat capacity at constant pressure: 657 J/(kg·K) [5]
- Thermal conductivity: 4.2 W/(m·K) [5]
- Elasticity matrix (Voigt notation): [6]

$$\mathbf{D} = \begin{bmatrix} D_{11} & D_{12} & D_{13} & D_{14} & D_{15} & D_{16} \\ D_{12} & D_{22} & D_{23} & D_{24} & D_{25} & D_{26} \\ D_{13} & D_{23} & D_{33} & D_{34} & D_{35} & D_{36} \\ D_{14} & D_{24} & D_{34} & D_{44} & D_{45} & D_{46} \\ D_{15} & D_{25} & D_{35} & D_{45} & D_{55} & D_{56} \\ D_{16} & D_{26} & D_{36} & D_{46} & D_{56} & D_{66} \end{bmatrix} = \begin{bmatrix} 418 & 210 & 156 & 0 & -10 & 0 \\ 210 & 343 & 231 & 0 & 29 & 0 \\ 156 & 231 & 394 & 0 & -33 & 0 \\ 0 & 0 & 0 & 222 & 0 & 59 \\ -10 & 29 & -33 & 0 & 104 & 0 \\ 0 & 0 & 0 & 59 & 0 & 169 \end{bmatrix} \times 10^9 \text{ Pa.}$$

Ultrafast transmission electron microscopy

The time-resolved three-dimensional experiments were performed in an ultrafast transmission electron microscope (a modified JEOL TEM 2100) operated at 200 kV, using a hybrid pixel detector (CheeTah T3, Amsterdam Scientific Instruments). The sample was excited with a 1030 nm wavelength laser (Tangerine, Amplitude Systems) with a 300 fs pulse duration. The pump laser was focused to a 200 μm (FWHM) spot with an average incident fluence of 5 mJ/cm^2. A 35-degree slanted, aluminum-coated mirror with a reflectivity of approximately 50% was placed above the sample plane to generate a transient laser grating on the sample. The sinusoidal grating pattern with a periodicity of 1 μm arose from the interference between the direct pump beam and its reflection from the slanted face of the mirror. The vanadium dioxide lamella was excited at a repetition rate of 12 kHz, ensuring that the sample returned to its monoclinic ground state between subsequent laser pulses. The specimen was mounted on a double-tilt holder, allowing precise adjustment of the tilt angles α and β.

Electron probe pulses were generated through photoemission from a guard ring LaB_6 cathode

excited by 258 nm laser pulses. Further details of the experimental setup can be found in Ref.[7] The temporal width of the electron bunches was approximately 1.5 ps, retrieved from photon-induced near-field electron microscopy measurements. The time delays between the pump-probe pulses were controlled using a motorized delay stage between the pump and probe beam path.

For the 4D STEM scans, we used the second smallest condenser aperture, with a diameter of 50 μm, to generate a more parallel electron beam with a convergence angle of a few mrad. This configuration allowed us to separate individual diffraction peaks. The electron beam brightness was fine-tuned to optimize the balance between resolution in diffraction space and imaging. The total exposure time per delay and pixel was 30 s, with an average electron flux of 100,000 electrons per 30 seconds incident on the direct electron detector. This exposure time corresponds to signal accumulation over many repeated stroboscopic pump–probe cycles at a repetition rate of 12 kHz rather than a single continuous dynamic event. Experiments were performed at room temperature, with measurements taken along one of the main zone axes in the [01-1] projection of the monoclinic structure. TEM images of the same regions before and after extended measurements show no new defects, dislocations, or permanent contrast changes. Repeated measurements on the same and different regions yield consistent results, indicating that the observed strain, bending, and contrast dynamics arise from reversible photoinduced processes.

The dataset consists of $21 \times 8 = 168$ measurement points in real space, with each point containing a 2D diffraction pattern acquired with a 30-second exposure time. A total of 15 4D STEM sets were collected, corresponding to 15 time delays, leading to a total acquisition time of approximately 21 hours. The nominal spatial resolution was 160 nm, determined by the pixel spacing, though the actual resolution was limited by the electron beam spot size. The effective beam spot size on the specimen was estimated by comparing a sharp specimen image with a reconstructed virtual STEM image. The electron beam spot size, determined

from an error function fit to the edge spread function of a sharp edge, was approximately 400 nm (Fig. S3).

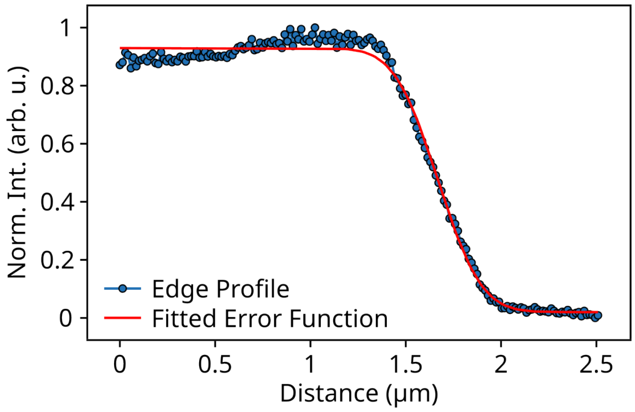

Figure S3: **Beam size estimation from an error fit to the edge spread function.**

Ultrafast 4D STEM data analysis

The experimental data was analyzed as follows: For each time delay and each pixel in real space, a corresponding diffraction pattern was obtained. Background subtraction was performed using the difference of Gaussians method using the Pyxem software[8] to remove the inelastic background and thermal diffuse scattering near the zero-order diffraction peak. The diffraction patterns were also corrected for salt-and-pepper noise. To ensure consistency across time delays, the total intensity of all counts in real space was normalized, such that the sum of counts over all pixels was conserved across all time delays. All diffraction patterns were aligned with respect to each other. Time-resolved virtual images were generated by applying a virtual mask to the diffraction patterns in Py4DSTEM[9] across all pixels in real space and for all time delays.

Raw data

Figure S4 presents representative nano-beam electron diffraction patterns recorded at a fixed real-space position for increasing pump–probe delays. With increasing delay, the M1 superstructure reflections progressively weaken and eventually disappear, consistent with the

photo-induced suppression of the monoclinic lattice distortion.

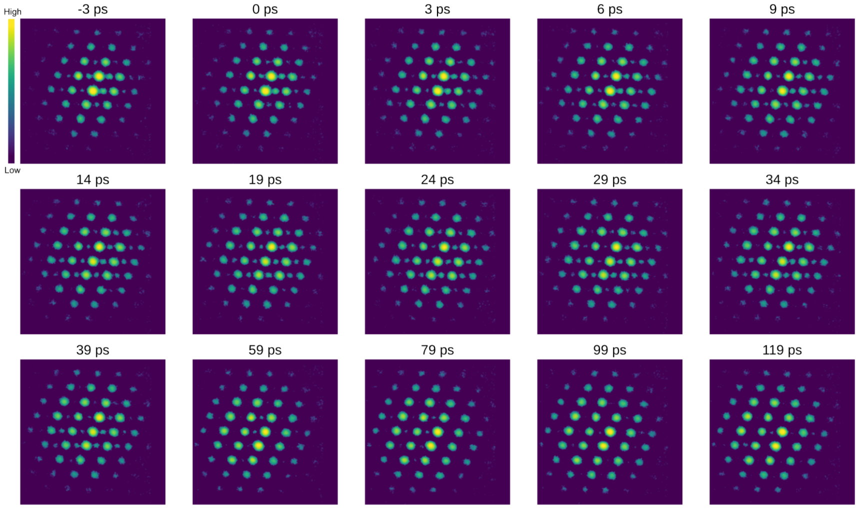

Figure S4: **Ultrafast 4D STEM.** Diffraction patterns as a function of the pump-probe delay are shown for a single position in real space.

Ultrafast strain mapping

Strain analysis was conducted using the py4DSTEM software[9] . The strain at different time delays was referenced to negative delays by using the same reciprocal lattice vectors. At negative delays, the total strain was set to zero following a median strain approach. The work flow of strain analysis is shown in Fig. S5. Due to symmetry considerations of the structured light excitation, we analyze in the main text only the strain along the x-direction, ε_{xx}, which is perpendicular to the generated optical grating fringes.

The strain values are extracted from changes in reciprocal lattice vector positions (Bragg peak centroids) rather than from diffraction intensities. Under strong optical excitation, temperature-induced lattice vibrations (Debye–Waller effect) reduce Bragg peak intensities through increased atomic mean-square displacements. However, this effect does not alter reciprocal lattice spacings and therefore does not introduce a systematic shift in peak positions. Since our strain analysis is based solely on peak positions, the Debye–Waller effect does not systematically influence the extracted strain values.

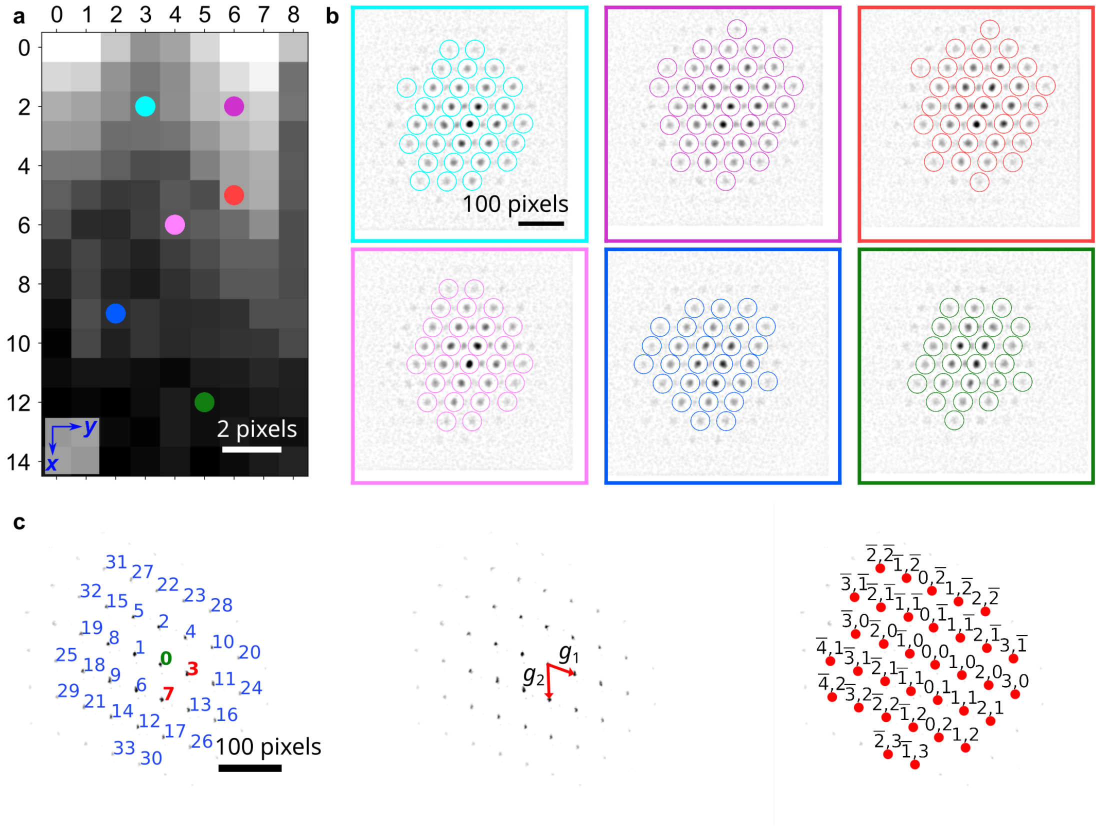

Figure S5: **Strain analysis procedure using ultrafast nanobeam electron diffraction.** (a) Several pixels of the virtual image for a given time delay are selected to generate a template for strain analysis. The corresponding diffraction patterns along the [01-1] zone axis are shown in (b). Diffraction peaks are identified for strain analysis. The M1-exclusive diffraction spots are disregarded due to their low intensity. (c) The diffraction peaks are labeled, and the reciprocal lattice vectors are chosen. Deviations from these reciprocal lattice vectors indicate strain.

Other strain components

To provide a complete picture of the lattice response during the phase transition, we additionally present the strain component ε_{yy} (Fig. S6). The structural phase transition occurs throughout the optically excited region and therefore affects both lattice directions.

The magnitude of ε_{yy} is, however, significantly smaller than that of ε_{xx} due to crystallographic orientation and the anisotropic lattice distortion of the M1-to-rutile transition. In our experimental geometry, ε_{xx} is predominantly aligned with the monoclinic a-axis, which undergoes the largest relative change (approximately 1%) across the transition.[10] By contrast, ε_{yy} represents a projection involving both the a- and c-axes, with the c-lattice parameter changing substantially less (approximately five times smaller than a[10]). Consequently, the projected strain amplitude along the y-direction is reduced.

As a result, periodic strain modulations associated with the transient grating are clearly resolved in ε_{xx}, while the corresponding oscillatory component in ε_{yy} is strongly suppressed and approaches the experimental noise level. This reduced visibility reflects crystallographic projection effects rather than the absence of phase-transition-induced lattice dynamics.

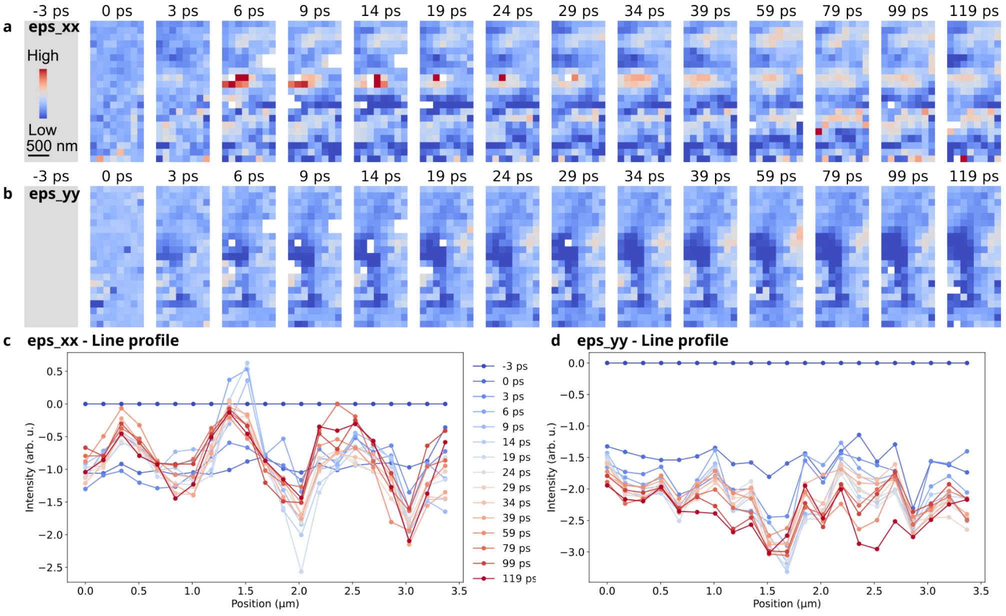

Figure S6: **Strain tensor components $\varepsilon_{xx}, \varepsilon_{yy}$ and the corresponding line profiles.**

Correlation of strain with virtual BF and DF imaging

Fig. S7 compares line profiles of virtual bright-field (VBF), virtual dark-field (VDF), and the extracted strain ε_{xx}, along with their correlations.

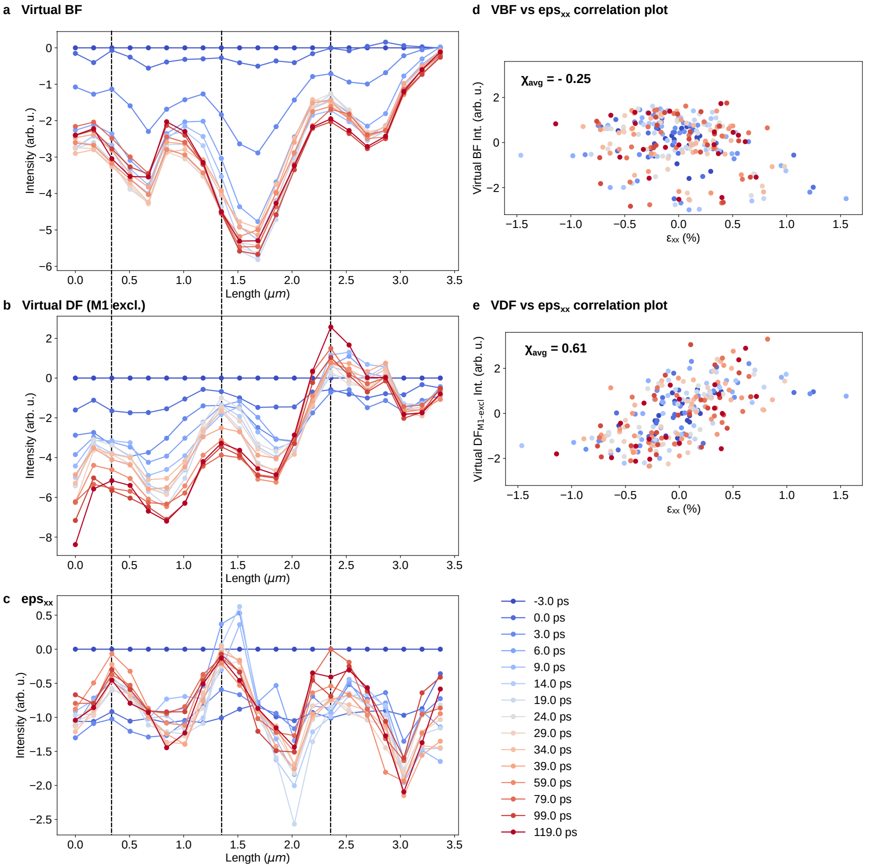

Figure S7: **Comparison of line profiles of virtual bright-field, virtual dark-field and $\varepsilon_{\mathbf{xx}}$ imaging and their correlations.** All quantities were extracted from a single ultrafast 4D STEM scan, ensuring identical temporal sampling and spatial registration across the virtual imaging modes and the strain analysis.

In Fig. S7e, the strong positive correlation between the VDF signal associated with the M1 Bragg reflections and the extracted strain ($\chi \approx 0.61$) indicates that both observables probe the same underlying structural order parameter. The VDF intensity directly reflects the presence and strength of the monoclinic lattice distortion, while the strain maps quantify the accompanying lattice deformation. Their in-phase spatial modulation therefore confirms that the measured strain is intimately linked to the structural phase transition.

In contrast, Fig. S7d shows the weak and negative correlation between the VBF signal and the strain ($\chi \approx -0.25$), highlighting the fundamentally different contrast mechanism of bright-field imaging. VBF contrast is sensitive to a convolution of effects, including diffuse scattering, local lattice disorder, and sample bending or tilts. These contributions can respond differently to the structural distortion associated with the phase transition, leading to a reduced and phase-shifted correlation with the strain. As a result, although VBF also exhibits spatial modulation following the transient grating excitation, its contrast is not phase-locked to the structural order parameter.

Taken together, these correlation values quantitatively demonstrate that diffraction-based virtual dark-field imaging and strain mapping provide selective, structure-sensitive information, whereas bright-field contrast mixes multiple scattering channels and is therefore less suitable for directly tracking the microscopic coupling between strain and the structural phase transition.

Reproducibility of strain mapping

To assess the reproducibility of the strain dynamics, we performed the same measurements on a separate lamella prepared under identical conditions. This lamella was selected independently from the region shown in the main text and exhibits comparable diffraction quality. Figure S8a presents the extracted in-plane strain maps ϵ_{xx} as a function of pump–probe delay. A clear spatial modulation of the strain is observed, with a periodicity that matches the imposed optical transient grating, demonstrating that the strain response follows the structured excitation profile. The static offset was subtracted from each time-dependent strain frame, highlighting the spatial modulation. In contrast to the analysis in Fig. 4, where the Bragg basis vectors were referenced to the first time delay, they were here referenced to the mean Bragg vector at each time delay so that the average strain over the selected 4D STEM area is zero.

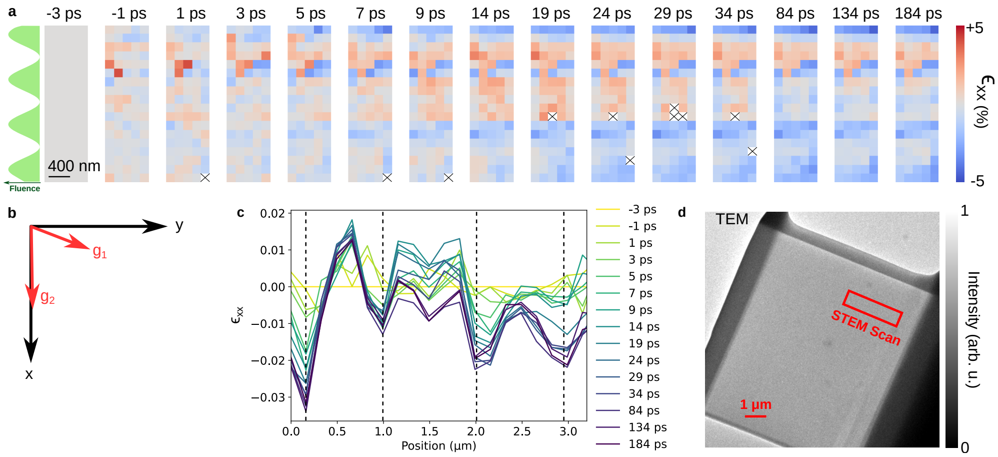

Figure S8: **Additional strain mapping data on a different lamella.** (a) In-plane strain maps as a function of time delay, showing spatial modulation following the optical grating. (b) Measurement orientation in real and diffraction space. (c) Line profiles of the strain integrated perpendicular to the optical grating direction, revealing periodic oscillations consistent with the structured excitation. The dashed lines are a guide to the eye. (d) TEM image of the lamella used for the second strain measurement.

Figure S8c shows line profiles integrated perpendicular to the optical grating direction for all time delays, revealing pronounced oscillations with the same spatial periodicity as the optical interference pattern. These measurements confirm that the spatially modulated strain response, governed by the excitation geometry, is reproducible across distinct sample regions. Figure S8d shows a TEM image of the second lamella, indicating the region of the STEM scan.

Time0 determination

In our experiments, the time0 between the laser pump and electron probe is determined by monitoring the intensity of diffraction spots that are exclusive to the M1 monoclinic phase of VO_2. By integrating the intensity of these M1-specific diffraction peaks as a function of pump-probe delay (Fig. S9), the time at which the signal begins to change indicates the temporal overlap of the pump and probe pulses. This approach provides a reference for the onset of photoinduced structural dynamics, as the reduction in intensity of M1-exclusive spots corresponds directly to the transition toward the rutile phase. At negative pump-probe

delays, the M1 diffraction intensity remains constant over repeated scans, demonstrating that the sample fully relaxes back to the monoclinic phase between successive pump pulses and that no cumulative heating or irreversible modification occurs during the stroboscopic measurements.

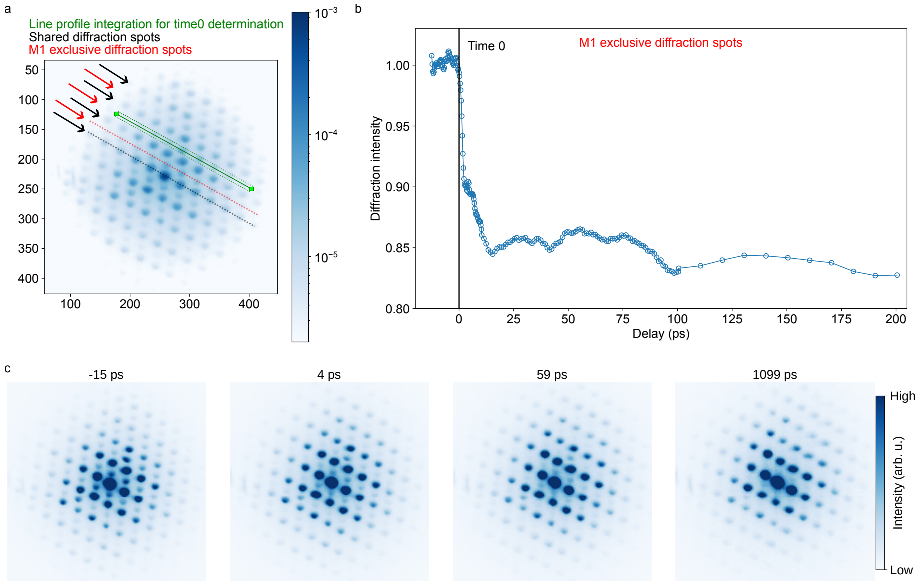

Figure S9: **Experimental determination of time0.** (a) Diffraction pattern of VO_2 in the monoclinic phase. (b) Integration over a line profile of M1-exclusive diffraction spots allows for a robust determination of time0 in an ultrafast transmission electron microscope. (c) Electron diffraction patterns at indicated time delays.

References

(1) Sasaki, H.; Watanabe, A. A New Growing Method for VO2 Single Crystals. *Journal of the Physical Society of Japan* **1964**, *19*, 1748–1748.

(2) Aramaki, S.; Roy, R. Single-crystal growth of VO2 by isothermal flux-evaporation. *J Mater Sci* **1968**, *3*, 643–645.

(3) Park, J. H.; Coy, J. M.; Kasirga, T. S.; Huang, C.; Fei, Z.; Hunter, S.; Cobden, D. H. Measurement of a solid-state triple point at the metal–insulator transition in VO2. *Nature* **2013**, *500*, 431–434, Number: 7463 Publisher: Nature Publishing Group.

(4) Polyanskiy, M. N. Refractiveindex.info database of optical constants. *Sci Data* **2024**, *11*, 94, Publisher: Nature Publishing Group.

(5) Kizuka, H.; Yagi, T.; Jia, J.; Yamashita, Y.; Nakamura, S.; Taketoshi, N.; Shigesato, Y. Temperature dependence of thermal conductivity of VO2 thin films across metal–insulator transition. *Jpn. J. Appl. Phys.* **2015**, *54*, 053201, Publisher: IOP Publishing.

(6) Dong, H.; Liu, H. Elastic properties of VO2 from first-principles calculation. *Solid State Communications* **2013**, *167*, 1–4.

(7) Ji, S.; Piazza, L.; Cao, G.; Park, S. T.; Reed, B. W.; Masiel, D. J.; Weissenrieder, J. Influence of cathode geometry on electron dynamics in an ultrafast electron microscope. *Structural Dynamics* **2017**, *4*, 054303.

(8) Cautaerts, N.; Crout, P.; Ånes, H. W.; Prestat, E.; Jeong, J.; Dehm, G.; Liebscher, C. H. Free, flexible and fast: Orientation mapping using the multi-core and GPU-accelerated template matching capabilities in the Python-based open source 4D-STEM analysis toolbox Pyxem. *Ultramicroscopy* **2022**, *237*, 113517.

(9) Savitzky, B. H. et al. py4DSTEM: A Software Package for Four-Dimensional Scanning Transmission Electron Microscopy Data Analysis. *Microsc Microanal* **2021**, *27*, 712–743.

(10) Kucharczyk, D.; Niklewski, T. Accurate X-ray determination of the lattice parameters and the thermal expansion coefficients of VO2 near the transition temperature. *Journal of Applied Crystallography* **1979**, *12*, 370–373, _eprint: https://onlinelibrary.wiley.com/doi/pdf/10.1107/S0021889879012711.
